# Adrenal Crisis Masquerading as Sepsis in a Patient With Autoimmune Multimorbidity: A Diagnostic Challenge on the Acute Medical Ward

**DOI:** 10.7759/cureus.99607

**Published:** 2025-12-19

**Authors:** Bola Reyad, Hogir Aldawoody

**Affiliations:** 1 General Internal Medicine Department, St. Mary's Hospital, Isle of Wight NHS Trust, Isle of Wight, GBR; 2 Emergency Department, St. Mary's Hospital, Isle of Wight NHS Trust, Isle of Wight, GBR

**Keywords:** adrenal crises, antiphospholipid syndrome (apls), immunocompromised, long-term steroid, sepsis

## Abstract

A patient in her mid-60s with autoimmune multimorbidity, including cutaneous lupus, Sjögren’s syndrome, antiphospholipid syndrome (APLS), and adrenal insufficiency, presented with a five-day history of fever, vomiting, diarrhea, and profound fatigue. Adrenal crisis typically occurs in individuals with adrenal insufficiency during periods of physiological stress such as infection, dehydration, surgery, or inadequate steroid dosing, and should be suspected when patients develop hypotension, gastrointestinal upset, electrolyte disturbances, or fail to improve with standard therapy. Because these symptoms overlap significantly with sepsis, diagnostic confusion is common, especially in acutely unwell immunosuppressed patients, where infection is frequently presumed. Sepsis itself presents with fever, raised inflammatory markers, hypotension, and generalized malaise, mirroring many manifestations of adrenal crisis and often leading to initial misclassification.

Despite correctly applying steroid sick-day rules, which involve doubling usual glucocorticoid doses during acute illness, patients may still decompensate and require escalation to parenteral emergency doses. In this case, empirical antibiotics were started for presumed sepsis after investigations revealed raised C-reactive protein (CRP) and mild hypokalemia with no clear infectious focus. However, the rapid improvement following fluid resuscitation and adjusted steroid therapy highlighted adrenal crisis as the true underlying cause. This case emphasizes the importance of maintaining a high index of suspicion for adrenal crisis in any acutely ill patient with known adrenal insufficiency, ensuring timely stress-dose steroid administration before pursuing alternative differential diagnoses.

## Introduction

Adrenal crisis is a rare but potentially fatal complication of adrenal insufficiency that requires prompt recognition and management. Chronic adrenal insufficiency may be primary, due to adrenal gland destruction; secondary, due to pituitary impairment; or tertiary, resulting from prolonged exogenous steroid use. Its incidence is relatively low, but patients remain at lifelong risk of adrenal crisis, with common features including hypotension, severe fatigue, abdominal pain, nausea, vomiting, electrolyte abnormalities (particularly hyponatremia and hyperkalemia), and hypoglycemia. These manifestations often resemble systemic infections, especially sepsis, making timely differentiation essential. Symptoms such as hypotension, vomiting, and fatigue often overlap with those of sepsis or gastroenteritis, complicating early diagnosis [1]. This diagnostic overlap is particularly challenging in patients with autoimmune multimorbidity who are immunosuppressed and receiving chronic corticosteroids.

Although both adrenal crisis and sepsis may present with circulatory collapse, fever, gastrointestinal symptoms, and elevated inflammatory markers, several distinguishing features can aid early recognition. Adrenal crisis more commonly causes refractory hypotension despite fluids, unexplained hyponatremia, hypoglycemia, and absence of a clear infectious source, whereas sepsis is typically associated with fever, leukocytosis, evidence of organ dysfunction, and a suspected or confirmed infection.

Current Surviving Sepsis guidelines recommend early administration of stress-dose steroids in patients with known adrenal insufficiency who present with sepsis or septic shock, emphasizing that hydrocortisone should not be delayed when adrenal crisis is suspected [2]. Understanding normal physiology is essential: healthy adults produce approximately 5-10 mg/m²/day of cortisol, but during physiological stress, such as acute illness, trauma, or infection, cortisol production may increase three- to tenfold. Therefore, failure to meet these metabolic demands predisposes patients with adrenal insufficiency to rapid decompensation.

Even when patients adhere to “sick-day rules,” which recommend increasing oral steroid doses during physiological stress, decompensation may still occur. Clinicians must remain vigilant for subtle signs of adrenal crisis, especially when patients fail to respond as expected to standard sepsis management protocols [3].

## Case presentation

A 64-year-old patient presented with a five-day history of fever, profuse vomiting, watery diarrhea, and progressive fatigue. Her medical history included cutaneous lupus erythematosus (Ro/La positive), Sjögren’s syndrome, antiphospholipid syndrome (APLS), a prior left nephrectomy, obstructive sleep apnea managed with continuous positive airway pressure (CPAP), and adrenal insufficiency secondary to long-term steroid use (prednisolone 10 mg daily). She was also taking mycophenolate for her autoimmune disease.

Following standard sick-day rules, she had doubled her prednisolone dose to 20 mg in the morning and 10 mg in the evening, but continued to deteriorate. On arrival to the acute medical unit, she was afebrile but hypotensive (90/60 mmHg) and mildly tachycardic. She appeared clinically dehydrated yet remained alert. Given her autoimmune background and history of adrenal insufficiency, adrenal decompensation was strongly suspected. However, concurrent infection remained a possibility due to recent fever and gastrointestinal symptoms.

Empirical intravenous amoxicillin was started for presumed sepsis alongside intravenous crystalloids and continuation of the increased steroid regimen. Mycophenolate was temporarily withheld.

Investigations revealed a mildly elevated C-reactive protein (CRP) that peaked at 38 mg/L before normalizing, normal sodium levels, mild transient hypokalemia, and stable renal function (Table 1). Blood cultures, viral PCRs, and imaging, including CT pulmonary angiography and CT abdomen/pelvis, showed no evidence of infection. Fecal calprotectin was elevated at 179 μg/g, folate was low, and vitamin B12 results were pending at discharge. 

**Table 1:** Summary of key investigations CRP: C-reactive protein; eGFR: estimated glomerular filtration rate

Parameter	Day 1	Day 2	Reference Range	Units
CRP (Peak)	38	13	< 5	mg/L
Potassium (K⁺)	3.0	4.4	3.5–5.1	mmol/L
Sodium (Na⁺)	136	146	135–145	mmol/L
Creatinine	74	90	45–90	μmol/L
eGFR	>60	>60	>60	mL/min/1.73 m²
Hemoglobin	98	111	115–155	g/L
WCC	4.4	7.0	4.0–11.0	×10⁹/L
Platelets	167	257	150–450	×10⁹/L
Blood Sugar	6.4	7.1	3.5–7.8	mmol/L
Fecal Calprotectin	179	–	< 50	μg/g
Folate	2.8	–	4.6–18.7	μg/L

The patient showed significant clinical improvement within 48 hours of intravenous fluid resuscitation and continuation of steroid therapy. Her gastrointestinal symptoms resolved, inflammatory markers normalized, and she was discharged on a tapered steroid regimen (10 mg AM, 5 mg PM). A short course of oral amoxicillin and metronidazole was prescribed due to recent household exposure to *H. pylori*.

Follow-up with gastroenterology was arranged to assess the raised fecal calprotectin and low folate, and further monitoring with rheumatology, respiratory, and dermatology continued for her autoimmune conditions.

## Discussion

This case highlights the diagnostic dilemma of distinguishing adrenal crisis from sepsis, especially in patients with autoimmune disease on chronic corticosteroids. The initial presentation with fever, raised inflammatory markers, and gastrointestinal upset suggested infection; however, the absence of a positive microbiological source and the rapid clinical improvement following intravenous fluids and steroid continuation supported adrenal crisis as the primary diagnosis.

Despite adherence to sick-day guidance, this patient experienced decompensation, consistent with literature indicating that oral dose-doubling may be insufficient during severe physiological stress [4,5]. Prompt escalation to parenteral hydrocortisone may be necessary in such settings.

The incidental findings of elevated fecal calprotectin and low folate may indicate underlying gastrointestinal pathology such as celiac disease or inflammatory bowel disease, which warrants further outpatient evaluation.

Clinicians should maintain a high index of suspicion for adrenal crisis when patients with known adrenal insufficiency present with sepsis-like symptoms but fail to respond to antibiotics. Early recognition and appropriate steroid replacement remain vital to prevent morbidity and mortality [6,7].

## Conclusions

This case demonstrates how adrenal crisis can mimic sepsis, particularly in autoimmune, immunosuppressed patients. Notably, the patient did not exhibit all classical features of adrenal insufficiency yet responded promptly to appropriate steroid therapy, highlighting that a full constellation of symptoms is not required to make the diagnosis. It underscores that adherence to sick-day steroid protocols may not always prevent crisis and emphasizes the importance of reviewing initial assumptions when patients fail to improve. A negative infection workup in the context of hypotension and electrolyte disturbance should raise suspicion for adrenal insufficiency. Multidisciplinary follow-up is essential to manage autoimmune comorbidities and prevent recurrence.
